# Hospital Specialist Palliative Care Team Influence on End-of-Life Care in Coronavirus Disease 2019? A Retrospective Observational Cohort Study

**DOI:** 10.1089/pmr.2022.0041

**Published:** 2022-10-21

**Authors:** Tony Duffy, R. Andrew Seaton, Alistair McKeown, Paul Keeley, Natalie Sanzone, Leza Quate, Eoghan Farmer, Harrison Stubbs

**Affiliations:** ^1^ St Columba's Hospice Care, Edinburgh, United Kingdom.; ^2^ Queen Elizabeth University Hospital, NHS Greater Glasgow and Clyde, Glasgow, United Kingdom.; ^3^ Glasgow Royal Infirmary, NHS Greater Glasgow and Clyde, Glasgow, United Kingdom.; ^4^ St Andrew's Hospice, Airdrie, United Kingdom.; ^5^ Inverclyde Royal Infirmary, NHS Greater Glasgow and Clyde, Glasgow, United Kingdom.

**Keywords:** COVID-19, hospital care, terminal care

## Abstract

**Objectives:**

The coronavirus 19 disease (COVID-19) pandemic has led to a renewed focus on end-of-life care. The majority of COVID-19 deaths occur in hospital, with patients cared for by generalists and hospital specialist palliative care teams (HSPCTs). This project aims at exploring the potential influences of HSPCTs on end-of-life care in COVID-19.

**Methods:**

A retrospective observational study was carried out by exploring four end-of-life care themes in a Scottish hospital population who died from COVID-19. Comparison was made between cohorts seen by HSPCTs versus generalist clinicians.

**Results:**

Analysis of 119 patients across NHS Greater Glasgow and Clyde (NHSGGC) health board demonstrated that COVID-19 patients seen by HSPCTs were more likely to be younger (median 77 vs. 81 years; *p* = 0.02), have a cancer diagnosis (21.7% vs. 5.4%; *p* = 0.01), die sooner after admission (median four vs. six days; *p* < 0.01), and be commenced on a syringe driver (89.1% vs. 42.5%; *p* < 0.01). Differences detected across four end-of-life care themes comparing HSPCTs with generalist teams were minimal with documentation and prescribing in keeping with available guidance.

**Conclusion:**

Consistencies in end-of-life care observed across NHSGGC cohorts draw attention to the potential wider impact of HSPCT roles, including education, guideline development, and mentoring. Understanding such diverse effects is important to support funding and development of HSPCTs. Further research is required to better quantify the impact and heterogenous influences of HSPCTs in general.

## What Is Already Known

Coronavirus 19 disease (COVID-19) has led to increased exposure of generalist clinicians to end-of-life care.

Hospital specialist palliative care teams (HSPCTs) provide patient care and a supportive role for generalists.

## What This Study Adds

End-of-life care in hospital settings may be safely standardized with the support of HSPCT support.

Established palliative care practices appear to be transferrable to COVID-19 disease.

## How This Study May Affect Research

Further research is required to quantify the impact of hospital palliative care teams on patient outcomes and generalist care.

## Introduction

A significant proportion of patients in Scotland will have access to hospital specialist palliative care team (HSPCT) review at end-of-life as in-patients.^1^ There remains a need for more evidence to support the effectiveness and positive impact of HSPCTs for patients, families, and staff.^2^ In particular, there is a need for more evidence around HSPCT roles in non-malignant conditions.^2^ The rapidly changing landscape of hospital end-of-life care (EOLC) that occurred with the coronavirus 19 disease (COVID-19) pandemic provided an opportunity to explore differences in EOLC delivered by hospital generalist teams compared with HSPCTs.

With this in mind, we conducted a retrospective comparative cohort study comparing two cohorts of patients who died from COVID-19 disease in the NHS Greater Glasgow and Clyde (NHSGGC) health board area of Scotland: one cohort looked after by generalist health care professionals and one that received HSPCT review. By comparing four areas of EOLC, this study aims at answering the question:

“Do hospitalized patients who die from COVID-19 disease receive different end-of- life care if reviewed by the hospital specialist palliative care team?”

## Methods

### Study design

A retrospective comparative cohort design was selected to ensure that only hospitalized patients who died with a confirmed positive polymerase chain reaction (SARS-CoV-2 PCR) test were included. Cohorts were divided as follows:

*Cohort 1*: patients who died with COVID-19 disease reviewed by an HSPCT

*Cohort 2*: patient who died with COVID-19 disease who were managed by generalist teams

### Participants

The EOLC data were collected from a sample of 140 NHSGGC hospital patients who died with COVID-19 between April 1 and April 12, 2020. This timeframe was selected, as it represents a crucial period when NHSGGC hospital admissions reached the peak of the pandemic and COVID-19 EOLC guidance in Scotland had recently been introduced.

Inclusion criteria were as follows: (1) patients older than the age of 18 who died in hospital; (2) who had a confirmed +ve SARS-CoV-2 PCR test within 28 days of death; (3) who received face-to-face HSPCT or generalist care at ward level and had complete admission notes and medication prescription charts scanned onto the NHSGGC Portal system.

Exclusion criteria were as follows: (1) patients with equivocal SARS-CoV-2 PCR testing or X-ray diagnosis only; (2) patients who died in intensive care units (ICU); or (3) patients with incompletely scanned clinical notes on the NHSGGC Portal system.

The HSPCT phone advice was not included in this study. This is not always recorded in the patient's notes and can be difficult to differentiate from other specialty reviews if not identified as HSPCT advice. Patients in ICU and psychiatry settings were excluded due to complexities around obtaining complete patient documentation across different IT systems.

### Data collection

Data collection occurred on NHSGGC computer systems using the PORTAL digital patient record platform. Encrypted patient community health index (CHI) numbers were supplied and overseen by a Caldicott Guardian. A data collection tool and protocol were developed using Microsoft Excel and distributed to data collectors to standardize data collection and reduce observer bias.

Population data, including age, sex, and source of admission, were collected to build study demographics. Admission date, date of first SARS-CoV-2 +ve PCR, and date of death were included as reference point variables to calculate multiple time indices. The EOLC ward setting was broken down into medical, surgical, care of the elderly (COTE), infectious diseases (ID), and high dependency/coronary care units (HDU/CCU). A standardized assessment of the underlying health of the study population was performed using an online Charlson co-morbidity index (CCI) calculator.^3^ A selection of common co-morbidities was included for further analysis. Date and time of death was confirmed using the digitally scanned medical certificate of death.

### End-of-life care themes

Four EOLC themes were selected based on NHSGGC guidance at end of life (GAEL) advice for health care professionals that outlines important aspects of EOLC.^4^ A synopsis of how each section of the GAEL guidelines relates to the four EOLC themes is provided in Table 1

**Table 1.** Selection of Four End-of-Life Variables Using Guidance at End-of-Life Guidelines as a Framework

Selected study themes	Related GAEL guidance^4^
ACDs	“Informative, timely and sensitive communication is an essential component of each individual patient's care.”
DNACPR	“An objective of DNACPR policy is to encourage and facilitate open, appropriate and realistic discussions with patients/relatives/carers/POA in the context of agreed goals of care. All discussions and decisions must be clearly documented.”
CSCI medication use	“Significant decisions about a patient's care including diagnosing dying, are made on the basis of multi-disciplinary discussion- consider the need for a subcutaneous infusion of medication via a syringe pump”
PRN medication use	“Scottish palliative care guidelines anticipatory prescribing”

ACD, advance care discussion; CSCI, continuous subcutaneous infusion; DNACPR, do not attempt cardiopulmonary resuscitation; GAEL, guidance at end of life; POA, power of attorney; PRN, “Pro re nata” use of medication when symptoms arise.

1. Advance care discussion (ACD): the term “advance care discussion” was employed to incorporate documented evidence of the following: (1) a discussion with the patient/relative/power of attorney (POA) relating specifically to the diagnosis of COVID-19 disease, including prognostic implications and treatment options; (2) that may also include exploration of preferred place of care/death (home, care home, hospice, hospital) or discussion about escalation to higher levels of care (HDU/ICU) and palliative care approaches.
2. Do not attempt cardiopulmonary resuscitation (DNACPR) documentation: evidence of DNACPR discussion was confirmed if both of the following were found: (1) a completed DNACPR form scanned onto PORTAL applicable at time of patient death and (2) documented evidence of a discussion between a senior health care professional and patient/relative/POA about the rationale behind decisions to complete a DNACPR form. Pre-admission DNACPR forms were not included unless a review of this decision was evident in the context of a COVID-19 diagnosis.
3. Continuous subcutaneous infusion (CSCI) dosage and timing of initiation before death: the maximum titrated dose (in milligrams) of the following medications in each CSCI was recorded: Morphine; Oxycodone; Alfentanil; Midazolam; Levomepromazine; Haloperidol; and Hyoscine butylbromide. Doses were recorded from CSCI prescription sheets. These are the most commonly used medications for end-of-life symptom relief in NHSGGC. The selection is comparable with other EOLC studies of COVID-19 disease. The start date and time of CSCI were recorded to investigate the timing of CSCI use around patient death.
4. As required (PRN) end-of-life symptom medication use: the cumulative dose (in milligrams) administered in the last 48 hours of life for each of the following medications was recorded: Oral Morphine equivalent; Oral Oxycodone equivalent; Alfentanil; Midazolam; Levomepromazine; Haloperidol; and Hyoscine Butylbromide. As both morphine and oxycodone can be given as either oral or subcutaneous doses, both were converted to their oral equivalencies. A ratio of subcutaneous/oral equivalency of 2:1 was used for both.

### Data analysis

Univariate analysis was performed on all data. Frequency counts were generated for nominal and ordinal variables (*n*; %). Interval-ratio variables, descriptive statistics were presented for all interval-ratio variables as median and interquartile ranges (IQRs).

Mann–Whitney *U* (MW) testing was used to test for differences between the cohort data in interval-ratio variables. Pearson Chi-square (CHI^2^) testing was applied to binary/nominal variables. Both tests were analyzed using 0.05 as the cut-off level of significance.

IBM SPSS (IBM, Inc., Chicago, IL) version 26 was used to perform the statistical tests.

## Results

Complete data were collected for 119 (85%) of the 140 patients in the sample group. Twenty-one (15%) patients were excluded from final collection. This consisted of eight patients who died in the ICU and 13 who died in a psychiatric setting. There were 46 (38.7%) patients in the HSPCT cohort, with 73 (61.3%) in the general care cohort. Demographics and co-morbidities are summarized in Table 2.

**Table 2.** Cohort Demographics and Co-Morbidities

Cohort	HSPCT	General care	
Group total (*n*)	46	73	Significance
Age, years; median (IQR)	77 (71:82)	81 (75:84)	***p* = 0.02 MW**
Sex male: female	26:20 (56.5% male)	48:25 (65.8% male)	*p* = 0.20 CHI^2^
Co-morbidities, *n* (%)			
COPD	16 (34.7)	15 (20.5)	*p* = 0.08 CHI^2^
Hypertension	23 (50)	39 (53.4)	*p* = 0.71 CHI^2^
CKD	10 (21.7)	17 (23.2)	*p* = 0.84 CHI^2^
Dementia	10 (21.7)	24 (32.8)	*p* = 0.19 CHI^2^
LVSD/CCF	9 (19.5)	18 (24.6)	*p* = 0.51 CHI^2^
Diabetes			
Type 1	0	3 (4.1)	*p* = 0.16 CHI^2^
Type 2	12 (26)	14 (19.1)	*p* = 0.37 CHI^2^
No known co-morbidities, *n* (%)	7 (15.2)	7 (9.5)	*p* = 0.35 CHI^2^
CCI score, median (IQR)	5 (4:8)	5 (5:6)	*p* = 0.55 MW
Cancer diagnosis, *n* (%)	10 (21.7)	4 (5.4)	***p* = 0.01 CHI^2^**
None	36	69	
Colon	1	1	
Brain primary	1	0	
Prostate	3	2	
Melanoma	0	1	
Lung	3	0	
Hematological	2	0	
Metastatic disease	2	0	*p* = 0.07 CHI^2^
Admission source, *n* (%)			
Own home	34 (73.9)	60 (82.2)	*p* = 0.28 CHI^2^
Care home	10 (21.7)	12 (16.4)	*p* = 0.47 CHI^2^
Residential care	1 (2.2)	1 (1.4)	*p* = 0.74 CHI^2^
Hospital transfer	1 (2.2)	0	*p* = 0.21 CHI^2^
Ward type, *n* (%)			
Medical	36 (78.3)	46 (63)	*p* = 0.08 CHI^2^
COTE	8 (17.4)	21 (28.8)	*p* = 0.16 CHI^2^
Surgical	1 (2.2)	2 (2.7)	*p* = 0.85 CHI^2^
Infectious diseases	1 (2.2)	0	*p* = 0.21 CHI^2^
HDU/CCU	0	4 (5.5)	*p* = 0.11 CHI^2^
Admission to death, days (IQR)	4 (2:7)	6 (4:17)	***p* ≤ 0.01 MW**
Positive SARSCoV2 PCR until death, days (IQR)	4 (2:8)	5 (3:7)	*p* = 0.38 MW

Underscored statistical tests are demonstrating statistically significant differences.

CCF, congestive cardiac failure; CCU, coronary care units; CHI^2^, Pearson Chi-square; CKD, chronic kidney disease; COPD, chronic obstructive pulmonary disease; COTE, care of the elderly; HDU, high dependency unit; HSPCT, hospital specialist palliative care team; IQR, inter-quartile range; LVSD, left ventricular systolic dysfunction; MW, Mann–Whitney *U* test; PCR, polymerase chain reaction; SARS-CoV-2, the virus causing COVID-19 disease.

The median age of HSPCT patients was lower at 77 years compared with an age of 81 years in those receiving general care (*p* = 0.02 MW). There was a small predominance of male patients in both cohorts, with a male-to-female ratio of 26:20 (56.5% Male) in the HSPCT cohort and 48:25 (65.8% Male) in the general care cohort.

No statistically significant difference was found in the prevalence of pre-existing patient co-morbidities. The CCI scores were similar between both cohorts with an HSPCT median of 5 and a general care median of 5 (*p* = 0.55 MW).

Only 14 patients had a confirmed cancer diagnosis, and these patients were more likely to have HSPCT review (21.7% vs. 5.4%, *p* = 0.01 CHI^2^). The CCI scores were similar between both cohorts with an HSPCT median of 5 and a general care median of 5 (*p* = 0.55 MW).

The time between hospital admission, SARS-CoV-2 positivity, patient death, and first HSCPT review is presented in Table 3.

**Table 3.** Hospital Specialist Palliative Care Team Admission Timeline

Admission to HSPCT review, days median (IQR)	2 (1:5)
Positive PCR to HSPCT review, days median (IQR)	2 (1–5)
HSPCT review to death, days median (IQR)	1 (0.75–2.25)

Documented evidence of an ACD was found in 42 (91.3%) of the HSPCT cohort and 68 (93.2%) of those receiving general care (*p* = 0.71 CHI^2^). Every patient had a DNACPR form in place when they died. DNACPR discussions occurred earlier after admission in patients seen by an HSPCT. The median duration was zero days (day of admission) compared with one day in the general care cohort. This did not meet the criteria for statistical significance (*p* = 0.06 MW). Full analysis is presented in Table 4.

**Table 4.** Advance Care Discussion (Do Not Attempt Cardiopulmonary Resuscitation)

Cohort	HSPCT	General care	Significance
ACD discussion documented, *n* (%)			*p* = 0.71 CHI^2^
Yes	42 (91.3)	68 (93.2)	
No	4 (8.7)	5 (6.8)	
DNACPR discussion, *n* (%)			*p* = 0.93 CHI^2^
Yes, with documented discussion	42 (91.3)	67 (91.8)	
Yes, with no documented discussion	4 (8.7)	6 (8.2)	
Admission to first ACD discussion, median days (IQR)	0 (0:2)	2 (0:7)	*p* = 0.01 MW
Admission to DNACPR completion, median days (IQR)	0 (0:2)	1 (0:4)	*p* = 0.06 MW
Time from PCR+ to ACD discussion, median days (IQR)	1 (0:2)	1 (0:3)	*p* = 0.39 MW
Time from PCR+ to DNACPR discussion, median days (IQR)	0 (0:2)	1 (0:2)	*p* = 0.75 MW

Underscored statistical tests are demonstrating statistically significant differences.

A CSCI was commenced in 41 (89.1%) of the patients seen by HSPCTs compared with 31 (42.5%) of those receiving general care (*p* < 0.01 CHI^2^). Maximum CSCI doses reached for each of the seven selected EOLC medications were comparable across both cohorts. Results are presented in Table 5.

**Table 5.** Continuous Subcutaneous Infusion Use in Cohorts

Cohort	HSPCT	General care	Significance
CSCI use, *n* (%)			***p* ≤ 0.01 CHI^2^**
Yes	41 (89.1)	31 (42.5)	
No	5 (10.9)	42 (57.5)	
CSCI maximum dose, median mg (IQR)			
Morphine	15 (10:20)	10 (10:20)	*p* = 0.23 MW
Oxycodone	15 (6:20)	20 (20:20)	*p* = 0.50 MW
Alfentanil	0.8 (0.3–1))	0.6 (0.5–0.8)	*p* = 0.81 MW
Midazolam	13 (10:20)	10 (6:15)	***p* = 0.03 MW**
Levomepromazine	18 (8:38)	25 (5:25)	*p* = 1.0 MW
Haloperidol	2 (2:2)	None	
Hyoscine butylbromide	70 (50:100)	70 (60:100)	*p* = 0.81 MW
Hours before death CSCI commenced, median (IQR)	26 (12:48)	26 (15:42)	*p* = 0.81 MW

Underscored statistical tests are demonstrating statistically significant differences.

No statistically significant difference was detected between cohorts in cumulative doses or administration frequency of breakthrough medications in the final 48-hours of life. Results are presented in Table 6.

**Table 6.** Pro Re Nata Medication Use

Cohort	HSPCT	General care	Significance
Final 48 hours total PRN medication doses, median mg (IQR)			
Morphine	12 (8:20)	12 (5:20)	*p* = 0.83 MW
Oxycodone	10 (7:15)	5 (2:7)	*p* = 0.27 MW
Alfentanil	0.45 (0.3:0.7)	0.225 (0.2:0.3)	*p* = 0.15 MW
Midazolam	6 (5:14)	4.5 (2:9)	*p* = 0.08 MW
Levomepromazine	7.5 (5:22.5)	7.5 (2.5:25)	*p* = 1.0 MW
Haloperidol	1.8 (1:2.5)	6.5 (0.5:12.5)	*p* = 1.0 MW
Hyoscine butylbromide	20 (20:40)	20 (20:40)	*p* = 0.61 MW
Hours before death last PRN medication given, median (IQR)	4 (2:17)	7 (2:17)	*p* = 0.35 MW
Required breakthrough medication, *n* (%)			
Morphine	25 (54.3)	51 (69.8)	*p* = 0.09 CHI^2^
Oxycodone	4 (8)	2 (2.7)	*p* = 0.15 CHI^2^
Alfentanil	10 (21.7)	6 (8.2)	*p* = 0.04 CHI^2^
Midazolam	35 (76)	54 (73.9)	*p* = 0.80 CHI^2^
Levomepromazine	6 (13)	8 (10.9)	*p* = 0.73 CHI^2^
Haloperidol	2 (4.3)	2 (2.7)	*p* = 0.64 CHI^2^
Hyoscine butylbromide	11 (23.9)	18 (24.6)	*p* = 0.91 CHI^2^
Did not require breakthrough	5 (10.8)	8 (10.9)	*p* = 0.99 CHI^2^
Indication for last PRN given, *n*			
Dyspnea ± agitation	38	58	
Secretions	3	6	
Nausea	0	1	

A comparison between PRN medication requirement in patients who had a CSCI implemented and those without CSCI use is presented in Table 7.

**Table 7.** Pro Re Nata Medication Use in Patients Receiving Medication via Continuous Subcutaneous Infusion

	CSCI used	No CSCI used	Significance
PRN drug required in those receiving CSCI: *n*			
Morphine	45	27	*p* = 0.58 CHI^2^
Oxycodone	6	0	***p* =** **0.04 CHI^2^**
Alfentanil	12	4	*p* = 0.20 CHI^2^
Midazolam	56	33	*p* = 0.35 CHI^2^
Levomepromazine	10	4	*p* = 0.37 CHI^2^
Haloperidol	3	1	*p* = 0.55 CHI^2^
Hyoscine butylbromide	21	8	*p* = 0.13 CHI^2^
No breakthrough required	6	7	*p* = 0.26 CHI^2^
Last 48-hour dose PRN, median mg.			
Morphine	15	10	*p* = 0.09 MW
Oxycodone	9	None	
Alfentanil	0.350	0.225	*p* = 0.24 MW
Midazolam	6.5	4	***p* ≤ 0.01 MW**
Levomepromazine	7.5	12.5	*p* = 0.72 MW
Haloperidol	1	12.5	*p* = 0.18 MW
Hyoscine butylbromide	20	20	*p* = 0.11 MW

Underscored statistical tests are demonstrating statistically significant differences.

## Discussion

This study set out to address the question: “Do hospitalized patients who die from COVID-19 disease receive different end-of- life care if reviewed by the hospital specialist palliative care team?”

Patients reviewed by HPSCTs were younger than those cared for by generalist teams. The median age of HSPCT referrals was comparable with a previous study from NHSGGC that reported an increased patient median age during the pandemic compared with their pre-COVID HSPCT cohort (73–76 years).^5^ This study supports evidence reported in previous studies with regard to sex distribution. The HSPCT patient referrals for COVID-19 were less likely to be male (*p* = 0.20) than those receiving generalist care but more likely to be male when compared with pre-pandemic HSPCT cohorts.^5–7^

Co-morbidity levels were similar between cohorts. The HSPCT COVID-19 referral CCI scores were lower than those reported in previous studies.^5,7–9^ The comparison of six co-morbidities in this study demonstrated no statistically significant difference in prevalence between cohorts. The prevalence of patients with cancer was low across both cohorts; although HSPCTs still saw the majority of these patients, this is consistent with previous reports.^7,10^

A fall in referral rates of patients with cancer to HSPCTs during the pandemic has been consistently reported,^7,10^ It has also been reported that CCI scores in patients with COVID-19 referred to HSPCTs have been lower than pre-pandemic levels.^7^ The fall in cancer referrals to HSPCTs may account for this finding, as points in the CCI are attributed for cancer diagnoses.

The HSPCT patients died sooner after admission than in the generalized cohort, and the time involved with patient care was even shorter than reported in previous studies (1 day vs. 1.4–2.26 days).^7,10–13^ These findings coupled with the low proportion of cancer patients, lower CCI scores, and distribution of co-morbidities presented in this study support previously observed changes in HSPCT patient phenotype during the pandemic.

It is possible that the fall in cancer-related referrals was due to less patients with cancer attending hospital as a result of concerns that admission could lead to contracting COVID-19. Most patients in both cohorts were admitted from their own home and died in general medical wards. A higher proportion of patients from care home settings were referred to the HSPCT. There is some indication from this study that COTE teams were less likely to refer patients dying from COVID-19 to HSPCTs. This may be due to familiarity with EOLC management or attributable to reported differences in COVID-19 presentations in advanced age and dementia.^14^

Documented evidence of ACDs was widely identified across both cohorts. Discussions took place earlier after admission in patients referred for HSPCT input. It is not possible to attribute this directly to HSPCT intervention though, particularly when most of these discussions took place on the day of admission and the median time to HSPCT referral was two days. All study patients had a DNACPR form in place at the time of their death.

Documentation of DNACPR discussion was found in around 91% of patients in both cohorts. DNACPR discussions occurred earlier in the HSPCT cohort, but again it is not possible to attribute this to HSPCT interaction. The time between a PCR +ve result and documentation of these themes was short across both cohorts, suggesting that a COVID-19 diagnosis in itself may have acted as a trigger for discussion.

The benefits of early advance care planning along with DNACPR discussion across most disease states have been widely promoted.^15–17^ Despite the patient-centered foundations of these approaches in the context of COVID-19, their communication and methods of form completion have been areas of controversy. The concept of “blanket” DNACPR forms along with media reports about patients or relatives being asked to sign DNACPR orders added complexity to what can already be emotive discussions for all involved.^18^

In this study, it became apparent that such discussions frequently took place close to the patient's death, often on the same day. Although not formally recorded, the data collection team noted a significant proportion of discussions that had taken place via telephone. The evidence of the impact of such changes in practice during the pandemic on patients, families, and health care professionals themselves is still emerging but does appear to demonstrate increased risk of psychological harm, severe acute grief reactions, and prolonged grief disorder.^19–21^

The use of CSCI medication to control symptoms of COVID-19 at end-of-life was more common in patients reviewed by an HSPCT, and this is consistent with previous studies.^5,7^ Possible reasons for this difference include familiarity of HSPCTs with their use, recognition of efficacy for managing symptoms, and experience accrued day-to-day addressing COVID-19 symptoms. Clinician observational evidence from prior studies suggests that CSCI use can improve symptom burden in COVID-19; however, there is a lack of empirical outcome measurement and qualitative data in this area.^5,22^

The CSCI medication use in the generalist cohort was also common. Maximum titrated medication doses were similar across both cohorts apart from higher midazolam doses in those reviewed by HSPCTs. The reason for this difference may relate to the severity of HSPCT patient symptoms or familiarity of midazolam use within HSPCTs. The CSCI doses were comparable with the existing literature and safely fell within the National Institute of Clinical Excellence (NICE) guidelines and Scottish Palliative Care Guideline (SPCG) recommendations.^23,24^

The timing of CSCI initiation is important to allow adequate symptom control. The median time between CSCI initiation and death in both cohorts was 26 hours, which indicates that any potential benefits of this administration route would generally have been obtained. The 26 hours mean duration of CSCI delivery before death observed across both cohorts suggests that although statistically significant data demonstrated that patients in the HSPCT cohort died sooner after admission, the use of CSCI medications did not appear to hasten death.

The PRN medication requirements were similar across both cohorts. Doses used were comparable with previous studies and conformed with both SPCG and NICE guidance.^23,24^ The HSPCT cohort patients were less likely to require PRN morphine for breathlessness, which may be attributable to the higher doses of midazolam administered via CSCI. Alfentanil is used when patients have significant renal failure or have developed opioid toxicity with other opioids.^25^ The PRN alfentanil was prescribed more often in HSPCT patients and it may be that knowledge of this drug's applications was more prevalent among these teams.

There was no clear influence on PRN requirement related to CSCI use identified but without analyzing sequential changes in patient symptom burden there are no conclusions that can be drawn here. The PRN Oxycodone was required more often when a CSCI was in use, but numbers were small. Oxycodone is the second-line opioid choice in NHSGGC when morphine has not been tolerated.^23^

Prescribing practices in this study observed across both cohorts coupled with the effective pharmacological timing and use of dose ranges within recommendations are important and reassuring findings. The early collaboration of generalists and palliative care specialists in Scotland to produce rapid clinical guidelines for managing symptoms of COVID-19 at end-of-life was potentially a significant driver toward standardizing practice.^26^

The HSPCT experience was transposed from pre-pandemic EOLC and combined with real-life clinical experience gained in the early days of the pandemic to produce this guidance. Symptom management recommendations from the SPCG pre-pandemic EOLC work combined with their COVID-19 specific guidance provided NHSGGC clinicians with solid foundations of reference during a time of uncertainty. The authors postulate that HSPCT clinicians working in NHSGGC had an influential role on the overall management of patients dying from COVID-19. This was most likely achieved by extrapolating practice based on previous experience, helping develop COVID-19 specific advice, leading by clinical example on the wards, and signposting to guidance resources.

## Conclusions

This study presents the first known attempt to explore potential differences in EOLC themes for patients with COVID-19 between HSPCT and generalist delivered care.

Evidence is presented to support important themes emerging from previous COVID-19 literature. This includes a change in HSPCT patient co-morbidity, referrals closer to time of death, and shorter periods of HSPCT patient contact. New evidence is presented surrounding the timing and documentation of advance care and DNAPCR discussions in COVID-19.

The study presents important evidence that supports the safe and appropriate use of medications to manage end-of-life symptoms in COVID-19. Taken in conjunction with existing evidence, this provides further evidence that previously established palliative care end-of-life practices are transferrable to COVID-19 disease.

### Limitations

This study does not include patients who survived to discharge or were admitted to ICU, limiting application to patients who died in a ward setting. Critical care and community settings are under-represented in this study, leaving important contributions of HSPCTs unexplored.

This study lacks patient-reported outcome measures and qualitative analysis of patient, relative, and staff experiences. Although it gives insight into the temporality of discussions, support and prescribing it fails to expand on the impact of these themes. There is no exploration of non-pharmacological interventions or oxygen therapy, making it difficult to analyze prescribing data in a robust, holistic manner.

### Future research

This study highlights the need for ongoing research into patient and family experiences of HSPCT compared with generalist care across end-of-life care. Themes that could be expanded include the impact of communication approaches, holistic care, and differences in grief outcomes. We suggest employing patient-reported outcome measures and qualitative analysis to better quantify any observable differences and investigate the “bleed down” effects of HSPCT input on EOLC on other clinical teams.

## Abbreviations Used

ACD: advanced care discussion
CCF: congestive cardiac failure
CCI: Charlson co-morbidity index
CCU: coronary care units
CHI^2^: Pearson Chi-square
CKD: chronic kidney disease
COPD: chronic obstructive pulmonary disease
COTE: care of the elderly
COVID-19: coronavirus 19 disease
CSCI: continuous subcutaneous infusion
DNACPR: do not attempt cardiopulmonary resuscitation
EOLC: end-of-life care
HDU: high dependency unit
HSPCT: hospital specialist palliative care team
ICU: intensive care unit
ID: infectious diseases
IQR: inter-quartile range
LVSD: left ventricular systolic dysfunction
MW: Mann–Whitney *U* test
NHSGGC: National Health Service Greater Glasgow and Clyde (Health board)
NICE: National Institute of Clinical Excellence
PCR: polymerase chain reaction
POA: power of attorney
PRN: “Pro re nata” use of medication when symptoms arise
SARS-CoV-2: the virus causing COVID-19 disease
SPCG: Scottish Palliative Care Guidelines
